# The role of macrophages in the susceptibility of Fc gamma receptor IIb deficient mice to *Cryptococcus neoformans*

**DOI:** 10.1038/srep40006

**Published:** 2017-01-11

**Authors:** Saowapha Surawut, Thunnicha Ondee, Sujittra Taratummarat, Tanapat Palaga, Prapaporn Pisitkun, Ariya Chindamporn, Asada Leelahavanichkul

**Affiliations:** 1 Medical Microbiology, Interdisciplinary Program, Graduate School, Chulalongkorn University, Bangkok, Thailand; 2 Medical Sciences Program, Faculty of Medicine, Chulalongkorn University, Bangkok, Thailand; 3 Department of Microbiology, Faculty of Medicine, Chulalongkorn University, Bangkok, Thailand; 4 Department of Microbiology, Faculty of Science, Chulalongkorn University, Bangkok, Thailand; 5 Division of Allergy, Immunology, and Rheumatology, Department of Medicine, Faculty of Medicine, Ramathibodi Hospital, Mahidol University, Bangkok, Thailand; 6 Center of Excellence in Immunology and Immune-mediated Diseases, Department of Microbiology, Faculty of Medicine, Bangkok, Thailand

## Abstract

Dysfunctional polymorphisms of FcγRIIb, an inhibitory receptor, are associated with Systemic Lupus Erythaematosus (SLE). Cryptococcosis is an invasive fungal infection in SLE, perhaps due to the de novo immune defect. We investigated cryptococcosis in the FcγRIIb−/− mouse-lupus-model. Mortality, after intravenous *C. neoformans*-induced cryptococcosis, in young (8-week-old) and older (24-week-old) FcγRIIb−/− mice, was higher than in age-matched wild-types. Severe cryptococcosis in the FcγRIIb−/− mice was demonstrated by high fungal burdens in the internal organs with histological cryptococcoma-like lesions and high levels of TNF-α and IL-6, but not IL-10. Interestingly, FcγRIIb−/− macrophages demonstrated more prominent phagocytosis but did not differ in killing activity *in vitro* and the striking TNF-α, IL-6 and IL-10 levels, compared to wild-type cells. Indeed, *in vivo* macrophage depletion with liposomal clodronate attenuated the fungal burdens in FcγRIIb−/− mice, but not wild-type mice. When administered to wild-type mice, FcγRIIb−/− macrophages with phagocytosed *Cryptococcus* resulted in higher fungal burdens than FcγRIIb+/+ macrophages with phagocytosed *Cryptococcus*. These results support, at least in part, a model whereby, in FcγRIIb−/− mice, enhanced *C. neoformans* transmigration occurs through infected macrophages. In summary, prominent phagocytosis, with limited effective killing activity, and high pro-inflammatory cytokine production by FcγRIIb−/− macrophages were correlated with more severe cryptococcosis in FcγRIIb−/− mice.

Systemic Lupus Erythaematosus (SLE) is an autoimmune disease with multi-factorial pathogenesis, including genetic and environmental factors^1^,^2^. Fc gamma receptors (FcγRs) recognize the Fc portion of immunoglobulin G. This recognition results in immune activation or inhibition^3^,^4^,^5^, depending upon integrated signalling through different FcγRs. Interestingly, the Fc gamma receptor IIb (FcγRIIb) is the only inhibitory signalling receptor in the FcγR family in either the mouse or human^5^,^6^. *In vitro*, hyper-immune responses, due to FcγRIIb defects, have been demonstrated in several immune cells, including dendritic cells, macrophages and B cells^7^. FcγRIIb-deficient (FcγRIIb−/−) mice develop lupus nephritis spontaneously at 6 months and are currently used as a lupus mouse model^5^. In spite of the deficiency, FcγRIIb−/− mice show highly effective immune responses against several types of pathogens, including bacteria, plasmodia and mycobacteria^7^,^8^,^9^. The fungal immune responses of FcγRIIb−/− mice have been inadequately tested, despite the high susceptibility to several fungal infections in patients with SLE^10^. Indeed, a high prevalence of cryptococcosis among Chinese patients with SLE has been reported^11^, and FcγRIIb loss-of-function polymorphisms are commonly associated with SLE in Asian populations^12^,^13^,^14^,^15^. Because cryptococcosis is an important invasive fungal infection in SLE, we examined the immune responses of FcγRIIb−/− mice to *Cryptococcus neoformans, in vivo* and *in vitro*.

## Results

### Increased severity of cryptococcosis in Fc gamma receptor IIb deficient mice

Lupus nephritis in FcγRIIb−/− mice developed spontaneously, as determined by high urine protein (urine protein creatinine index: UPCI) and anti-dsDNA antibodies at 24 weeks (Supplementary Fig. S1B,C). Age-related lupus manifestations in FcγRIIb−/− mice thus allowed the exploration of 2 SLE phases, asymptomatic and symptomatic, as determined by proteinuria and anti-dsDNA antibodies, despite normal serum creatinine (Scr) in both age groups (Supplementary Fig. S1A).

**Figure 1:**
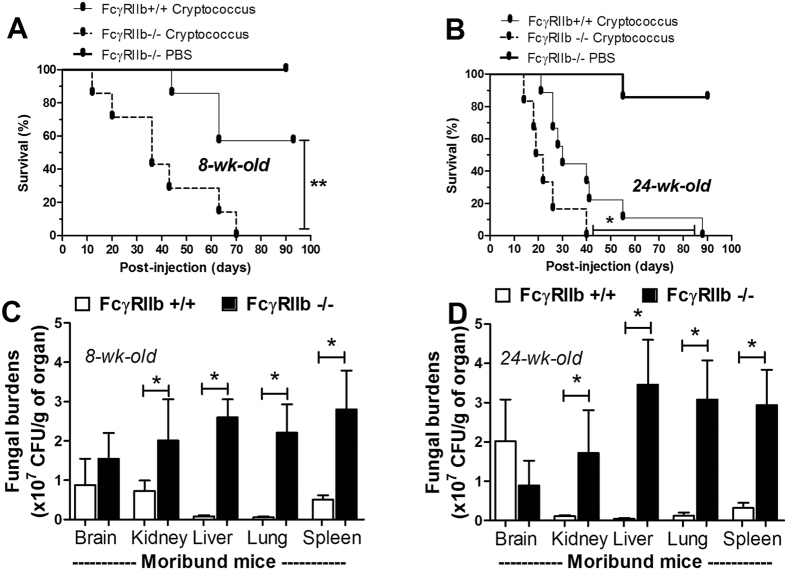
Survival analysis after *C. neoformans* administration in 8-week-old (n* *= 7/group: **A**) and 24-week-old mice (n* *= 7–9/group: **B**) are shown. In parallel, the fungal burdens in several internal organs of 8-week-old (n* *= 4–6/group: **C**) and 24-week-old mice (n* *= 5–6/group: **D**) are shown. The data are shown as the mean ± SE, and the presented values are combined from 2–3 independent experiments. *p < 0.05, **p < *0.01.*

**Figure 2:**
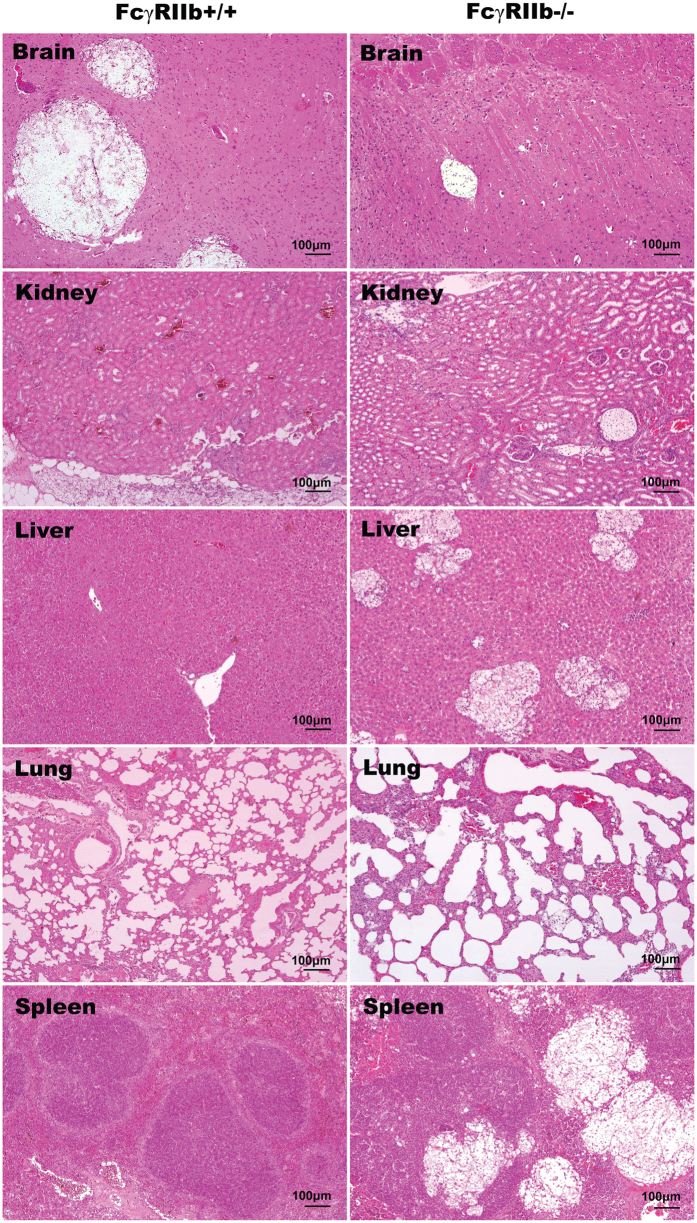
Representative histology with H&E (hematoxylin and eosin staining) at 100x magnification from 24-week-old mice in the moribund stage after *C. neoformans* administration. Cryptococcoma-like lesions were observed only in the brains of FcγRIIb+/+ mice (left column). In FcγRIIb−/− mice (right column), several internal organs had cryptococcoma-like lesions (brain, kidneys, liver, lung and spleen).

After *C. neoformans* administration, FcγRIIb−/− mice demonstrated higher mortality than the age-matched wild-type control mice in both the asymptomatic and symptomatic lupus groups (Fig. 1A–D). In the young mouse group, all of the FcγRIIb−/− mice, but only 57% of the wild-type mice, died or became moribund within 90 days of challenge (Fig. 1A). In contrast, all of the older mice with cryptococcosis died or became moribund by 40 and 90 days in the FcγRIIb−/− and wild-type groups, respectively (Fig. 1B). Interestingly, moribund FcγRIIb−/− mice, from both the young and old age groups, demonstrated higher fungal burdens and cryptococcoma-like-lesions in several internal organs, namely brain, kidney, liver, lung and spleen (Fig. 1C,D and Fig. 2). Conversely, in wild-type mice of both age groups, such lesions were found in only the brain, a major organ of infection, at the moribund stage (Fig. 2, Supplementary Fig. S2; only older mice shown).

**Figure 3:**
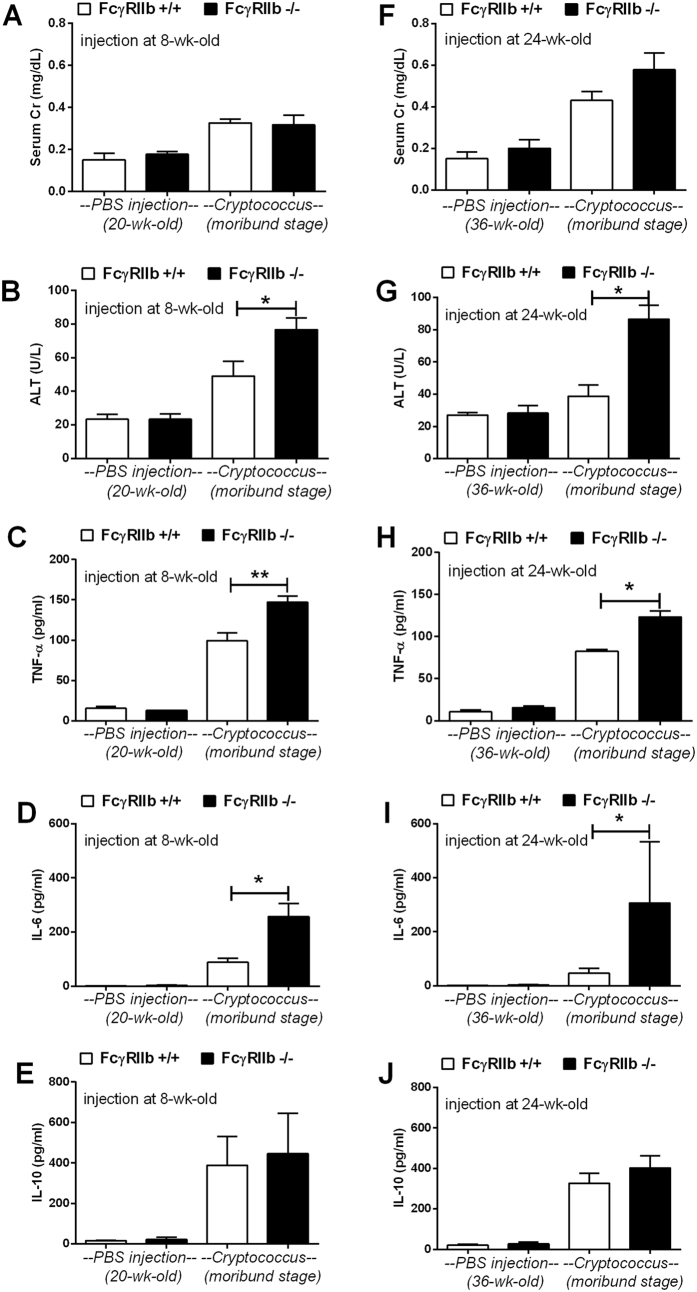
Organ injury and inflammatory cytokines at the moribund stage in 8-week-old (left column) (**A**–**E**) and 24-week-old mice (right column) (**F**–**J**), as demonstrated by serum creatinine (Scr), alanine transaminase (ALT), TNF-α, IL-6 and IL-10 levels. The data are shown as the mean ± SE, and the presented values are combined from 2 independent experiments (n* *= 4–5/group). *p < *0.05.*

**Figure 4:**
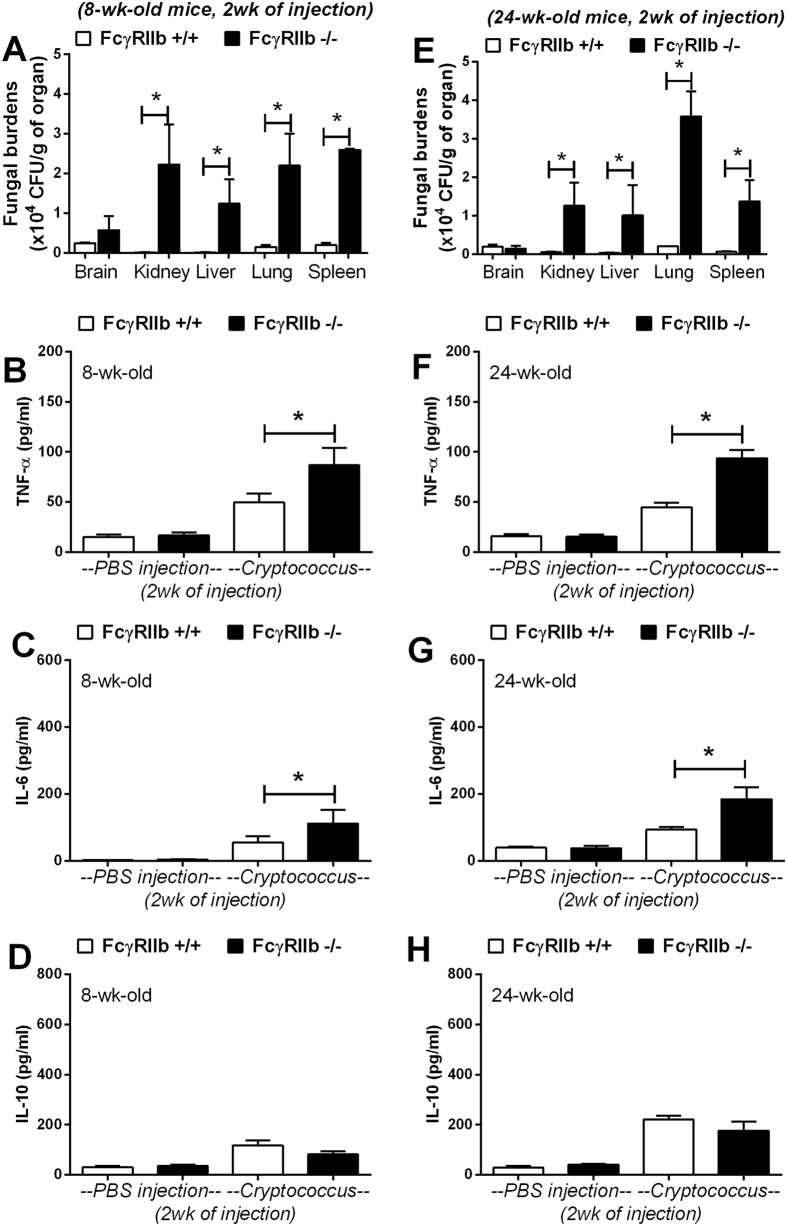
Fungal burdens in the internal organs of 8-week-old (left column) and 24-week-old mice (right column) at 2 weeks post-*C. neoformans* administration (**A**,**E**). Serum cytokines measured include TNF-α (**B**,**F**), IL-6 (**C**,**G**) and IL-10 (**D**,**H**). The data are shown as the mean ± SE, and the presented values are the combined from 2 independent experiments (n* *= 4–5/group). *p < *0.05.*

**Figure 5:**
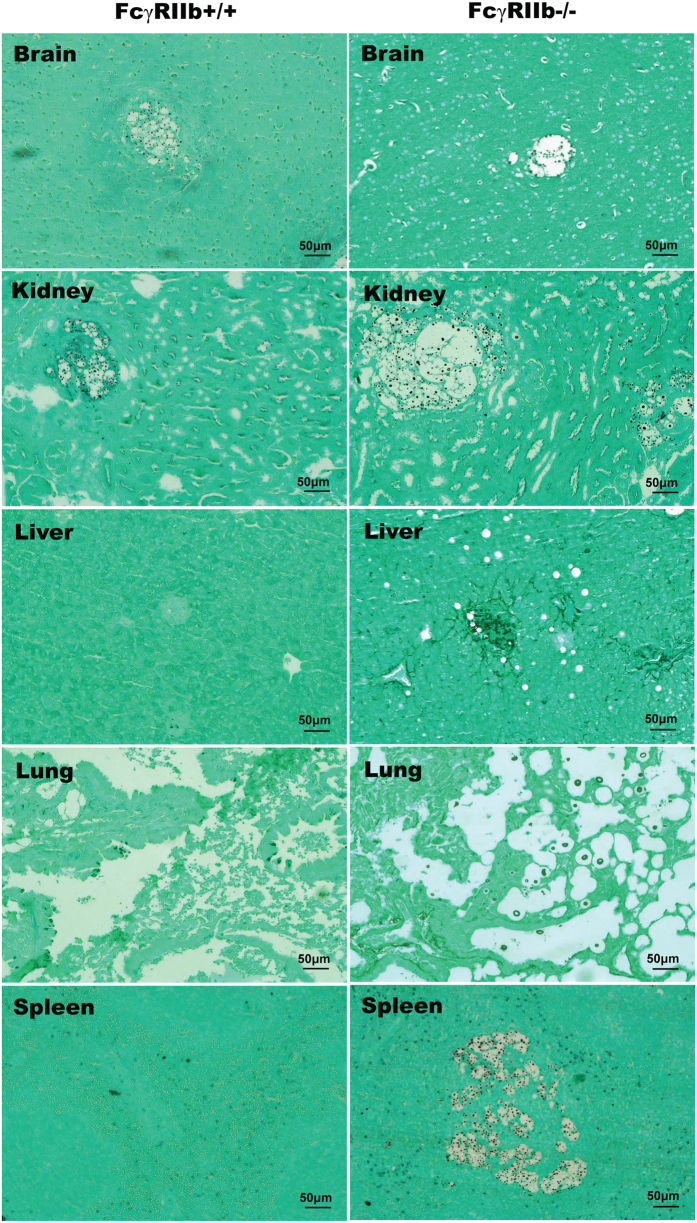
Organ histology with Grocott’s silver staining (GMS) at 200x magnification from 8 week-old mice at 2 weeks post-*C. neoformans* infusion, demonstrating cryptococcoma-like lesions in the brain and kidney of FcγRIIb+/+ mice (left column), and in several internal organs (brain, kidneys, liver, lung and spleen) of FcγRIIb−/− mice (right column).

At the moribund stage, cryptococcosis was more severe in both the young and old groups of FcγRIIb−/− mice. The levels of Scr, ALT and cytokines (TNF-α, IL-6 and IL-10) in wild-type were 0.33 ± 0.02 mg/dl, 49 ± 9 U/L and 99 ± 10, 89 ± 14 and 389 ± 142 pg/ml and in FcγRIIb−/− young mice were 0.32 ± 0.04 mg/dl, 77 ± 7 U/L, and 147 ± 8, 257 ± 48 and 446 ± 199 pg/ml (Fig. 3A–E). In parallel, these parameters in the old group of wild-type were 0.43 ± 0.04 mg/dl, 39 ± 7 U/L, and 82 ± 2, 47 ± 16 and 327 ± 50 pg/ml and in FcγRIIb−/− old mice were 0.58 ± 0.08 mg/dl, 87 ± 9 U/L and 123 ± 7, 307 ± 227 and 403 ± 59 pg/ml (Fig. 3F–J). After 2 weeks of cryptococcosis, the fungal burdens in the internal organs were higher in FcγRIIb−/− mice at the moribund stage (Fig. 4A,E). Cryptococcoma-like lesions were found in most organs in FcγRIIb−/− mice, but only in the brain and kidney in wild-type mice, in both age groups (Fig. 5, Supplementary Fig. S3; only 8-week mice shown). Anti-dsDNA antibody titer was slightly elevated in 2 out of 4 of 8-week-old FcγRIIb−/− mice (Supplementary Fig. S1), implying the beginning of SLE symptoms (incipient SLE) in this age group. The high susceptibility to fungi in these mice might due to ongoing SLE disease. When fungi were administered to younger FcγRIIb−/− mice, 4 weeks of age, none showed elevated anti-dsDNA antibodies, suggesting the absence of incipient SLE (Supplement Fig. S6). Fungal burdens in brain, lung and spleen, but not kidney and liver, were higher in 4-week-old FcγRIIb−/− mice compared with age-matched wild-type controls (Supplementary Fig. S6). Accordingly, the high susceptibility to cryptococcosis in the FcγRIIb−/− strain appeared to be due to the gene defect and less likely a result of autoantibody stimulation or ongoing SLE.

Additionally, the levels of pro-inflammatory serum cytokines, TNF-α and IL-6, but not an anti-inflammatory cytokine, IL-10, were higher in FcγRIIb−/− mice (Fig. 4B–D,F–H). Moreover, infected FcγRIIb−/− mice showed a tendency toward higher levels of total immunoglobulin (measured as total gamma globulin protein from protein electrophoresis) but this did not reach a statistically significant level (Supplementary Fig. S4). Therefore, cryptococcosis, either at 2 weeks or when moribund, was more severe in FcγRIIb−/− than wild-type mice, as demonstrated by higher fungal burdens in most internal organs, higher liver enzyme levels, and higher pro-inflammatory cytokine levels but not higher anti-inflammatory cytokine levels.

### FcγRIIb−/− macrophage responses to *C. neoformans*: prominent phagocytosis and pro-inflammatory cytokine production

**Figure 6:**
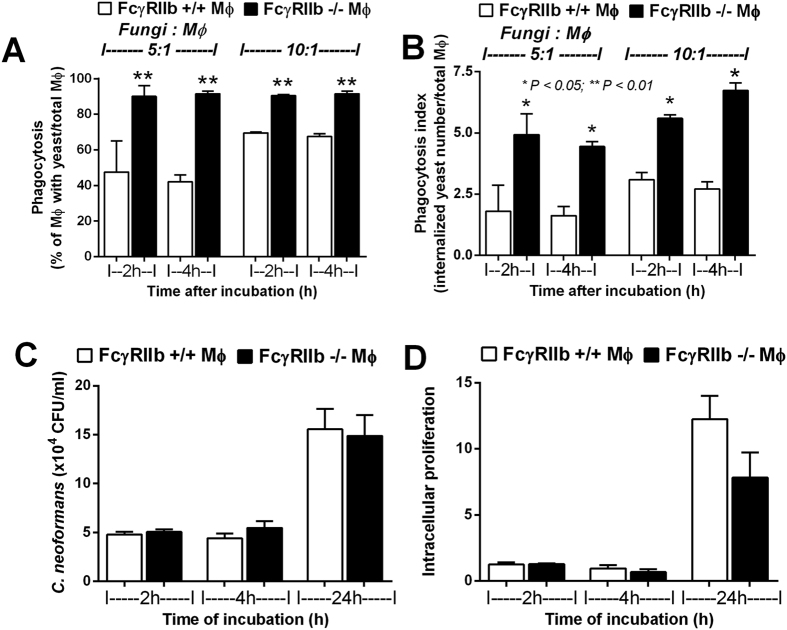
Percentage of macrophages (MФ) demonstrating phagocytosis (**A**) and the average number of phagocytosed fungi per MФ (total number of phagocytosed fungi/total MФ) after incubation with *C. neoformans* at ratios of fungi: MФ of 5:1 and 10:1 (**B**) are shown. The total killing ability (extruded and intracellular yeast) (**C**) and intracellular proliferation (concerning only intracellular yeast; see methods) (**D**) of MФ determined by the number of *C. neoformans* colonies after incubation with MФ from wild-type and FcγRIIb−/− mice are shown. *p < 0.05, **p < *0.01* (experiments were performed in triplicate).

Due to the importance of macrophages in fungal infection response and the presence of FcγRIIb in macrophages, we explored macrophage responses to *C. neoformans*. Interestingly, FcγRIIb−/− macrophages showed higher phagocytosis of *C. neoformans*. Macrophages were incubated with heat-killed *C. neoformans* in ratios of 5:1 and 10:1 (fungal cells to macrophages) for 2 h and 4 h (Fig. 6A). With the ratio of fungal cells to macrophages of 5:1, the percentages of macrophages with phagocytosed *C. neoformans* in wild-type and FcγRIIb−/− after 2 h incubation were 47.5 ± 17.5% and 90.1 ± 6.2%, respectively, and after 4 h incubation were 42.1 ± 4.2% and 91.5 ± 1.5%, respectively. At the ratio of 10:1, the percentages of phagocytosed macrophages in wild-type and FcγRIIb−/− after 2 h incubation were 69.5 ± 0.5% and 90.5 ± 0.5%, respectively, and after 4 h incubation were 67.5 ± 1.5% and 91.5 ± 1.5%, respectively.

Additionally, the average number of fungi in each macrophage was higher in FcγRIIb−/− cells, as determined by the phagocytosis index (number of internalized fungi/total macrophages) (Fig. 6B). In contrast, the macrophage killing activity, as determined by fungal viability after incubation with macrophages for 2, 4 and 24 h, was not different between wild-type and FcγRIIb−/− cells. The numbers of viable fungi in the macrophages with total killing ability (both extruded and intracellular yeasts were determined; see methods), *in vitro*, at 2, 4, and 24 h of fungal incubation with wild-type cells versus FcγRIIb−/− macrophages were 8.2 ± 0.8, 4.4 ± 0.5 and 5.5 ± 0.7 vs. 20.6 ± 2.8, 15.6 ± 2.1 and 14.9 ± 2.1 (x10^4^) CFU/ml, respectively (Fig. 6C). No difference in macrophage killing activity was observed using the intracellular proliferation assay. The intracellular proliferation assay that determined the viability of only intracellular yeasts also performed (see methods). Indeed, the intracellular proliferation amounts at 2, 4, and 24 h of fungi incubated with wild-type cells versus fungi incubated with FcγRIIb−/− macrophages were 1.3 ± 0.2, 1.0 ± 0.2 and 12.3 ± 1.8 vs. 1.3 ± 0.1, 0.7 ± 0.2 and 8.8 ± 1.9 units, respectively (Fig. 6D). Although a trend toward greater intracellular-killing by FcγRIIb−/− macrophages was observed, it was not statistically significant. The presence of macrophages did not reduce the colony count of fungi in the fungicidal activity assay, implying that cryptococci were viable, intracellularly.

**Figure 7:**
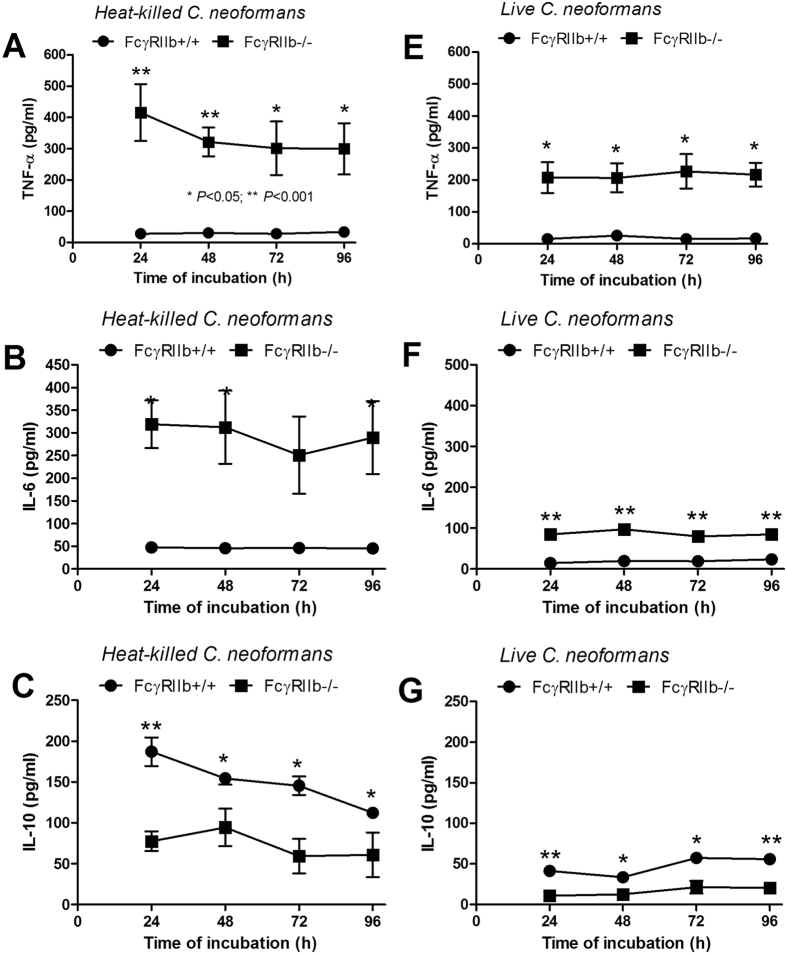
Cytokine levels (TNF-α, IL-6 and IL-10) in the supernatant media from macrophages of FcγRIIb+/+ or FcγRIIb−/− mice after activation with heat-killed (**A**–**C**) or live *C. neoformans* (**E**–**G**) are shown. *p < 0.05, **p < *0.01* (experiments were performed in triplicate).

In addition, the cytokine responses of FcγRIIb−/− macrophages to fungi were tested. FcγRIIb−/− macrophages, in response to heat-killed or live *C. neoformans*, produced higher levels of TNF-α and IL-6 but lower IL-10 levels (Fig. 7A–G), recapitulating the *in vivo* results (Figs 3 and 4).

### FcγRIIb−/− macrophages enhance the dissemination of cryptococci

**Figure 8:**
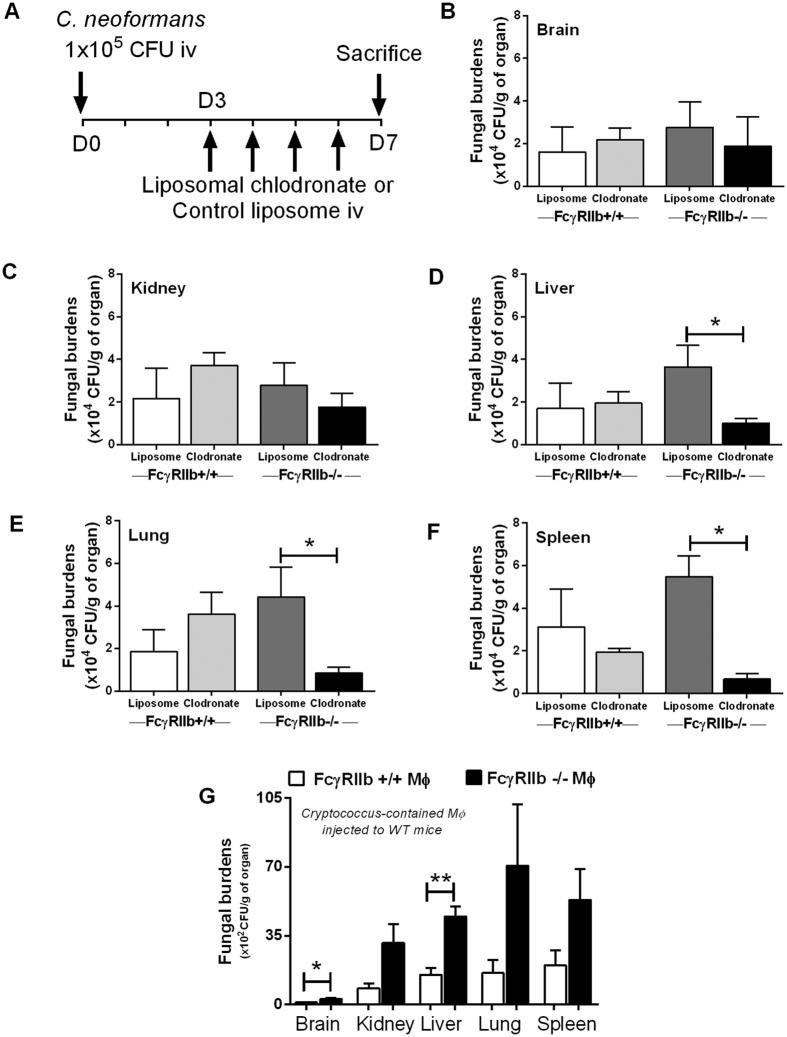
Timeline of a model for cryptococcosis in liposomal clodronate-induced macrophage depletion in 8-week-old FcγRIIb+/+ and FcγRIIb−/− mice (**A**) and fungal burdens in brain (**B**), kidney (**C**), liver (**D**), lung (**E**) and spleen (**F**) are shown (n* *= 4–5/group). Organ fungal burdens of FcγRIIb+/+ mice at 24 h after infusion of Cryptococcus-containing macrophages (infected macrophages) from FcγRIIb+/+ and FcγRIIb−/− mice are shown (**G**). (n* *= 4–5/group). Data are shown as the mean ± SE, and the presented values are combined from 2 independent experiments. *p < 0.05, **p < *0.01.*

*C. neoformans* is a facultative intracellular pathogen that demonstrates intracellular viability. Host phagocytes are used for fungal dissemination, and this is referred to as a “Trojan horse” mechanism^16^. Observing enhanced phagocytosis capacity but limited killing activity of FcγRIIb−/− macrophages, we hypothesized that FcγRIIb−/− macrophages may be responsible for more severe cryptococcosis *in vivo*. Accordingly, we tested cryptococcosis in FcγRIIb−/− and wild-type mice with liposomal clodronate-induced macrophage depletion (Fig. 8). Interestingly, macrophage depletion attenuated the fungal burdens in liver, lung and spleen in FcγRIIb−/− mice, but not in wild-type mice (Fig. 8). Fungal burdens at 7 days after fungal administration in the brain, kidney, liver, lung and spleen of FcγRIIb−/− mice with control (PBS) liposomes versus liposomal clodronate were 2.8 ± 1.2, 2.8 ± 0.9, 3.7 ± 1.0, 4.4 ± 1.4 and 5.5 ± 0.1 vs. 1.9 ± 1.4, 1.8 ± 0.6, 1.0 ± 0.2, 0.9 ± 0.3 and 0.7 ± 0.2 (x10^4^) CFU per organ weight (g), respectively (Fig. 8B–F). Subsequently, to determine if the high phagocytosis capacity of FcγRIIb−/− macrophages enhanced cryptococcal dissemination, we incubated yeast with wild-type and FcγRIIb−/− macrophages and administered the cells, with the phagocytosed cryptococcal cells, into wild-type mice. Indeed, the fungal burdens both in the brain, a major target organ of cryptococcosis, and in the liver, were higher in mice receiving *Cryptococcus*-containing FcγRIIb−/− cells (Fig. 8G). The fungal burdens in brain, kidney, liver, lung and spleen at 1 day after the administration of wild-type or FcγRIIb−/− macrophages were 1.1 ± 0.2, 8.3 ± 2.5, 15.1 ± 3.4, 16.1 ± 5.3 and 20 ± 7.7 vs. 2.9 ± 0.4, 31.4 ± 9.5, 44.8 ± 5.2, 70.7 ± 31.2 and 53.3 ± 15.7 (x10^2^) CFU per gram organ weight, respectively (Fig. 8G). Subsequently, the fungal burdens in the internal organs decreased due to the immunocompetence of wild-type mice (data not shown).

## Discussion

The FcγRIIb loss-of-function polymorphism is one of the important genetic associations of SLE, particularly in Asian populations^12^,^13^,^14^,^15^. Accordingly, FcγRIIb−/− mice, a lupus nephritis model, may be a useful mouse model of SLE in Asians. Our results suggest the possibility that the increased severity of cryptococcosis in FcγRIIb−/− mice is, at least in part, due to the unique properties of FcγRIIb−/− macrophages including enhanced phagocytosis and elevated pro-inflammatory cytokine responses.

Although intravenous administration is not the natural route of cryptococcal infection, which is transmission through pulmonary system, an intravenous model is adequate for a proof of concept of the difference between the two mouse strains. Indeed, FcγRIIb−/− mice showed more severe cryptococcosis than wild-type mice. The immune response against fungal infection is predominantly dependent on cell-mediated immune responses. The defect in FcγRIIb, the only inhibitory receptor in the FcγR family, induces an enhanced immune response and effectively controls several organisms^7^,^8^,^9^. FcγRIIb−/− mice were more susceptible to cryptococcosis, either in symptomatic or asymptomatic lupus, compared to the age-matched wild-type controls. There was also an age-dependent increase in the mortality rate for cryptococcosis. The mortality rate in wild-type mice at 8-weeks-old vs. 24-weeks-old was different (*p *= 0.015 by log-rank test, Fig. 1A,B), but there was no difference between the FcγRIIb−/− groups. The severity of cryptococcosis in FcγRIIb−/− mice was independent of the manifestation of lupus symptoms and age. These results support the susceptibility to cryptococcosis of patients with FcγRIIb polymorphisms, with either SLE or non-SLE^10^,^11^,^12^.

Subsequently, the severity of cryptococcosis was evaluated with different parameters at 2 weeks, the earlier stage of infection, and in the moribund stage. The 2 age groups, as expected, showed similar results. Disseminated cryptococcosis, including fungal organisms found in several internal organs, which was observed in FcγRIIb−/− mice, was similar to the disseminated cryptococcosis observed in patients with compromised immune systems^17^. However, the lesions were limited mostly to the brain in wild-type mice, a major target organ^18^, as usually observed in the immuno-competent human host. Surprisingly, despite the absence of generalized lesions in wild-type mice, the brain lesions were larger than in FcγRIIb−/− mice. This difference remains to be investigated. It seems that the neurotropism characteristic of *C. neoformans* is not apparent in the infection in FcγRIIb−/− mice because the organism could survive in any organ. However, in wild-type mice, *C. neoformans* may prefer to grow in the brain because of more suitable nutritional conditions^19^,^20^,^21^. Enhanced pathogenesis due to disseminated disease was also indicated by more severe liver injury in FcγRIIb−/− mice.

Enhanced cryptococcal dissemination in the FcγRIIb−/− mice underscores the role of this genetic lesion in pathogenesis. The inflammatory cytokines, TNF-α and IL-6, but not IL-10, were higher in FcγRIIb−/− than wild-type mice. This is a response to the higher fungal burden, in addition to the anti-inflammatory defect. Perhaps serum IL-10 is too low to balance out the pro-inflammatory immune responses, leading to more severe organ histopathology and symptoms. However, total immunoglobulin levels did not differ between the FcγRIIb−/− and wild-type mice after cryptococcal infection, despite the previous report of immunoglobulin hyperproduction in bacterial infection^8^. This inconsistency is possibly due to the difference in adaptive immune-response activated by these two organisms. Nevertheless, the beneficial effect of humoral immune response in controlling *C. neoformans* was demonstrated^22^,^23^,^24^.

Macrophages and T helper cells are the main immune cells responsible for the immune response to cryptococcosis^25^,^26^. FcγRIIb presents in macrophages but not in T cells^27^. Therefore, the higher fungal burdens in FcγRIIb−/− mice may be due to the primary defect in macrophages. Accordingly, we evaluated the phagocytosis activity, killing activity and cytokine responses of macrophages after exposure to *C. neoformans*. Interestingly, the phagocytosis of FcγRIIb−/− macrophages in response to cryptococci was elevated compared with wild-type cells, as reported for other organisms^8^,^9^. In FcγRIIb−/− mice, nearly all of the macrophages incubated with heat-killed *C. neoformans* phagocytosed approximately 6–7 yeasts per cell at 4 h (phagocytosis index). In contrast, approximately only 50–60% of wild-type macrophages phagocytosed yeast, and they did so with a reduced activity of 2–3 yeasts per cell. On the other hand, the total killing capacity and the intracellular proliferation activity of FcγRIIb−/− macrophages was not different from wild-type macrophages, unlike the responses to other organisms^8^, perhaps due to the immune evasion properties of *C. neoformans*^26^.

Cryptococcus is a facultative intracellular pathogen, which can utilize host macrophages to spread within the body, via the Trojan horse mechanism^16^. Cryptococci typically escape extracellular immune responses, survive and replicate intracellularly, transfer laterally between macrophages, and eventually invade tissue and organs^16^. They can use macrophages as trafficking vehicles for dissemination, particularly to pass through the blood-brain barrier into the central nervous system^28^. Interestingly, the depletion of macrophages, at least in certain situations, is associated with less severe pathogenesis^29^. We hypothesized that the elevated phagocytosis of FcγRIIb−/− macrophages and the immune evasion properties of *C. neoformans* enhanced the Trojan horse mechanism, resulting in more severe cryptococcosis *in vivo*. We tested cryptococcosis severity in a macrophage depletion model with daily liposomal clodronate injection in FcγRIIb−/− and wild-type mice. As expected, at 1 week after fungal administration, macrophage depletion led to lower fungal burdens in the liver, lung and spleen of FcγRIIb−/− mice, but not of wild-type mice. Nevertheless, liposomal clodronate not only depleted monocytes/macrophages but also reduced the numbers of dendritic cells and regulatory T cells^30^,^31^. Although loss of dendritic cells after liposomal clodronate injection might be responsible for the less severe cryptococcosis, the enhanced cryptococcosis severity after *Cryptococcus*-infected macrophage injection supports the greater pathogenic role of macrophages compared to dendritic cells. The inoculation of fungi-containing FcγRIIb−/− macrophages increased the fungal burdens in the brains and livers of wild-type mice at 24 h after administration. These results support the high phagocytosis capacity of FcγRIIb−/− macrophages and the enhancement of fungal transmission by macrophages, particularly through the blood-brain barrier. Together, these results support the importance of macrophages in cryptococcosis pathogenesis in FcγRIIb−/− mice and in patients with FcγRIIb loss-of-function polymorphisms.

In addition, the prominent pro-inflammatory cytokine response (TNF-α and IL-6), but not the anti-inflammatory cytokine (IL-10) response, was demonstrated in the FcγRIIb−/− groups, both *in vivo* and *in vitro*. This might be because glucuronoxylomannan (GXM), an important cryptococcal capsular polysaccharide, induces potent immunosuppression by direct engagement of FcγRIIb, an immunoinhibitory receptor, and stimulates greater IL-10 production^32^,^33^. In FcγRIIb−/− mice, perhaps GXM was unable to induce IL-10, resulting in the more severe pro-inflammatory cytokine storm and fungal sepsis. More studies on this topic are needed to explain the underlying mechanism.

Taken together, we conclude that more severe cryptococcosis in FcγRIIb−/− mice was due to enhanced dissemination, possibly through the Trojan horse mechanism, and the hyper-responsiveness of pro-inflammatory cytokine production during sepsis. This is the first report of the disadvantage of the prominent macrophage function of FcγRIIb−/− mice in cryptococcosis. In clinical translation, we propose FcγRIIb loss-of-function- polymorphisms as a new risk factor for cryptococcosis. Screening for FcγRIIb polymorphisms in patients with SLE, particularly in areas of endemic cryptococcosis, might be beneficial for patient management. Our results also implied the importance of the genetic-background differences among patients with SLE to micro-organism responses. The examination of the genetic background of individual patients with SLE might be clinically beneficial.

## Methods

### Animal models and *Cryptococcus neoformans* injection method

FcγRIIb−/− mice on the C57BL/6 background were kindly provided by Dr. Silvia Bolland (NIAID, NIH, Maryland, USA). The mice were originally constructed in a 129Sv/B6-hybrid background and were backcrossed onto the C57BL/6 background for 12 generations. Female C57BL/6 wild-type mice, age-matched to FcγRIIb−/− mice, were purchased from the National Laboratory Animal Center in Nakornpathom, Thailand. The animal protocols were approved by the Faculty of Medicine of Chulalongkorn University and followed NIH criteria. *C. neoformans* was isolated from a patient sample (Mycology Unit, King Chulalongkorn Memorial Hospital), identified by morphology, together with urease production and melanin synthesis (L-3,4-dihydroxyphenylalanine or DOPA test), and stored in Sabouraud dextrose Broth (SDB) at −80 °C. The sample accession process was approved by the Ethical Institutional Review Board, faculty of Medicine, Chulalongkorn University according to the declaration of Helsinki, with written informed consent. The same strain of *C. neoformans* was used in all of the experiments. Before use in experiments, *C. neoformans* was sub-cultured on Sabouraud dextrose agar (SDA) at 37 °C for 24 h. Asymptomatic (8-week-old without proteinuria) or symptomatic lupus (24-week-old with proteinuria) mice or age-matched wild-type control groups were injected, via tail vein, with 1 × 10^5^ yeast cells *C. neoformans* diluted in 200 μl of PBS. For survival analysis, the mice were observed for 90 days after fungal administration. The mice were sacrificed at the moribund stage, as determined by an inability to walk after touch stimulation. In other experiments, the mice were sacrificed at 2 weeks after fungal administration. At the time of euthanasia, blood was collected via cardiac puncture under isoflurane anaesthesia, and the internal organs (brain, lung, kidney, liver and spleen) were fixed with 10% formalin for histology or processed for fungal burden experiments (details below). In addition, to further investigate the importance of FcγR deficiency relative to autoantibody and incipient SLE status, 4-week-old FcγRIIb−/− mice and age-matched wild-type (non-significantly different anti-dsDNA antibody titers between both groups; Supplementary Fig. S6) were intravenously injected with *C. neoformans* at 1 × 10^5^ yeast cells and internal organ fungal burdens were determined at 2 weeks.

### *In vivo* macrophage depletion

Macrophage depletion with liposomal clodronate injection was performed in order to determine the role of macrophages in cryptococcosis, following a previously published protocol^28^. Female 8-week-old FcγRIIb−/− and wild-type mice were administered *C. neoformans* (yeast form) via the tail vein. Then, 200 μl/mouse liposomal clodronate (Encapsula Nanoscience, Nashville, TN, USA) (5 mg/ml) or control liposomes were injected to induce sustained monocyte depletion (Fig. 8A). The daily injections began on the third day of fungal administration and continued for 4 consecutive days. At 7 days post-inoculation, the mice were sacrificed and the internal organs were processed for fungal burdens and fixed in 10% formalin to confirm macrophage depletion (by immunohistochemical staining with an F4/80 antibody; Biolegend, San Diego, CA, USA). Macrophages were not detectable in organs after liposomal clodronate treatment in either FcγRIIb−/− or wild-type mice (Supplementary Fig. S5).

### Transfer of *Cryptococcus*-containing macrophages *in vivo*

Bone marrow (BM)-derived macrophages from FcγRIIb−/− and wild-type mice were allowed to phagocytose yeast cells and then they were infused into wild-type mice, as previously described^34^,^35^. Briefly, BM macrophages cultured in a 96-well plate at 2.5 × 10^4^ cells/well with 20% mouse serum were incubated with *C. neoformans* in the ratio of 5:1 (fungal cells to macrophages) for 2 h. The un-phagocytosed fungi were washed out with DMEM (3–5 washes). Subsequently, the macrophages were detached with cold-PBS washing (3–5 times) and centrifuged at 1,000 rpm for 10 min at 4 °C. Cell pellets were then resuspended with DMEM. The macrophages were counted and stained with trypan blue. Either FcγRIIb−/− or wild-type macrophages with internalized *Cryptococcus*, at 2.5 × 10^4^ cells, were intravenously administered to wild-type mice through the tail vein. The mice were sacrificed at 24 h and the fungal burdens determined.

### Fungal burdens and organ histology

To measure internal organ fungal burdens, the organs were weighed, homogenized, plated onto SDA and incubated at 37 °C for fungal colony enumeration at 48 h. For histology, the tissue samples were fixed in 10% formalin and embedded in paraffin; 4-μm sections were stained with haematoxylin and eosin colour (H&E) and Grocott’s silver stain (GMS) for *C. neoformans* identification. Quantitative measurement of the fungal infection area was performed by 2 blinded observers. Fields (10 selected randomly) were examined at 200x magnification, with the following criteria: 0, no fungi; 1, area of fungal infection <25%; 2, infected area involving 25–50% of the field; and 3, infected area ≥50% of the field.

### Blood chemistry, urine chemistry and cytokine analysis

Kidney injury was determined by serum creatinine (Scr) (QuantiChrom Creatinine Assay, DICT-500, BioAssay, Hayward, CA, USA), and liver injury was assessed via alanine transaminase levels (ALT) (EnzyChrom ALT assay, EALT-100, BioAssay). For the evaluation of antibody responses, mouse serum was analysed for total immunoglobulin^36^ by capillary protein electrophoresis (MINICAP-2 Sebia, Evry Cedex, France). The percentage of protein in the gamma zone of protein electrophoresis was converted into total immunoglobulin level by multiplying the ratio of protein at the gamma zone by the serum total protein. Serum total protein and urine protein were measured by Bradford protein assay. Urine protein creatinine index (UPCI), a representative of 24 h urine protein, was determined by the following equation; UPCI = spot urine protein/spot urine creatinine. Cytokine measurement (TNF-α, IL-6 and IL-10) in serum and supernatant media were measured using ELISA assays (eBioscience, San Diego, CA, USA).

### Anti-dsDNA antibody detection

Calf DNA (Invitrogen, Carlsbad, CA, USA) coated on 96-well plates was used for measuring anti-dsDNA antibodies, following a previously published protocol^37^. In brief, the plates were coated with calf DNA at 100 μg/well and incubated overnight at 4 °C. The plates were dried, filled with 100 μl/well of blocking solution, incubated at room temperature for 1.5 h and washed. Subsequently, mouse serum samples at 100 μl/well were added and incubated for 1 h. Then, the plate was washed, incubated with peroxidase-conjugated goat anti-mouse antibodies (BioLegend, USA) at 100 μl/well at room temperature for 1 h, washed and developed with ABTS peroxidase substrate solution (TMB Substrate Set; BioLegend) for 10 min in the dark. Finally, the stop solution (2 N H_2_SO_4_) was added, and the plate was read with a microplate photometer at a wavelength of 450 nm.

### Bone marrow-derived macrophage preparation

Bone marrow (BM)-derived macrophages were produced following an established protocol^38^. In brief, BM cells from femurs were centrifuged at 1,000 rpm in 4 °C for 10 min. Then, the cells were incubated in BMM or DMEM complete (DMEM supplement with 10% fetal bovine serum, 1% penicillin/streptomycin, HEPES and sodium pyruvate) plus 5% horse serum (HyClone^TM^ donor horse serum, Thermo Scientific, Waltham, MA, USA) and 20% L929-conditioned media in a humidified 5% CO_2_ incubator at 37 °C for 7 days. The cells were harvested at the end of the culture period using cold PBS, and the macrophage phenotype was confirmed by flow cytometry with anti-F4/80 and anti-CD11b antibodies (BioLegend, USA).

### *In vitro Cryptococcus neoformans*-induced macrophage cytokine production

Heat-killed *C. neoformans*, (immersion in a 60 °C water bath for 1 h) or live *C. neoformans*, at a dose of 5 × 10^5^ yeast cells/ml, were incubated with macrophages (1 × 10^5^ cells/well) in 96-well polystyrene tissue culture plates^39^. The culture supernatants were collected at various time points and stored at −80 °C until use. After the incubation, the cell viability was measured by the MTS cell proliferation assay (One Solution Cell Proliferation Assay, Promega Corporation, WI, USA) according to the manufacturer’s instructions^40^. Briefly, 20 μl of MTS were added to the culture plates, incubated for 2 h at 37 °C in a 5% CO_2_ incubator, and then read with a microplate photometer at a wavelength of 490 nm. All of the experiments showed cell viability of greater than 95% (data not shown).

### Phagocytosis, macrophage total killing activity and intracellular proliferation assays

The phagocytosis and macrophage killing activity assessments were performed according to a previous protocol, with slight modifications^34^,^35^. Briefly, BM-derived macrophages were added to 96-well plates at 2.5 × 10^4^ cells/well in DMEM complete and incubated overnight. After the incubation, LPS from *Escherichia coli* 026:B6 (Sigma-Aldrich, St. Louis, USA) was added at final concentration of 100 ng/ml and the plates were incubated in 5% CO_2_ at 37 °C for 24 h. The medium was then removed and 100 μl of complete DMEM with 20% normal mouse serum, as a source of opsonin, were added with heat-killed *C. neoformans* at various ratios of fungal cells to macrophages. These were incubated for various periods. After incubation, the wells were washed with 200 μl of PBS at least 3 times to remove un-ingested yeast and then the macrophages were detached with 200 μl of cold PBS. Then, the macrophages were transferred to a CytoSpin chamber (Thermo Scientific) and centrifuged at 600 rpm for 5 min to concentrate the cells into a single cell-layer for easier visualization. The macrophages were stained with Diff-Quick stain (Life Science Dynamic Division, Nonthaburi, Thailand). Macrophages containing yeast were counted as showing phagocytosis. At least 100 macrophages per well were counted. In parallel, the ingestion ability of each individual macrophage was determined as the average number of fungal cells in each macrophage (phagocytosis index), calculated as the total number of ingested fungi divided by the total number of macrophages. The phagocytosis activity was determined as the percentage of macrophages with phagocytosed cryptococci. All of the experiments were performed in triplicate.

Total cryptococcal cell killing activity was assessed using a previously published method which both extruded and intracellular yeasts were determined^34^. Briefly, BM-derived macrophages at 1 × 10^5^ cells/well were co-cultured with live *C. neoformans* at a ratio of 1:1 for 2, 4 and 24 h. LPS and mouse serum were used as mentioned above. After incubation, the culture supernatant was separated, and a lysis medium (distilled water containing 0.01% bovine serum albumin and 0.01% Tween-80) was added to the wells, for 20 min at 37 °C, to rupture the macrophage cell membranes. Then, the supernatant and the lysate were well mixed. Serial dilutions of the mixed lysates were plated on SDA for viable yeast colony forming unit (CFU) counts. Control cultures consisted of incubation medium alone plus *C. neoformans*. Macrophage total killing activity is inversely correlated with the number of yeast colonies. The intracellular and extracellular killing activity of macrophages was evaluated with this method.

Moreover, to determine only the intracellular killing activity, the intracellular proliferation assay was performed with methods that were slightly modified from those previous published^41^,^42^. Briefly, BM-derived macrophages, at 2.5 × 10^4^ cells/well, were incubated for 17 h with IFN-γ (10 ng/ml final concentration; BioLegend, USA). Then, LPS (100 ng/ml final concentration; Sigma-Aldrich) was added and incubation continued for 24 h. Cells were then washed with PBS. After that, live *C. neoformans*, at a ratio of 5:1, were added and incubated for 2 h in 20% normal mouse serum containing media to promote phagocytosis. After 2 h, the wells were extensively washed (4–5 times) to remove extracellular fungi. This was set as the 0 h time-point. For some of the culture wells at this time-point, macrophage cell lysis were induced by lysis medium and plated on SDA for the visualization of intracellular fungal viability for “phagocytosis activity at the 0 h time-point” for the further calculation. The remaining culture wells from the 0 h time-point were maintained in DMEM media at 37 °C, and the cells were subsequently lysed at 2, 4 and 24 h, for the determination intracellular fungal viability, as mentioned above. Because the difference in intracellular fungi might be due to the difference in phagocytosis activity at the 0 h time-point, the phagocytosis activity at the 0 h time-point was used for the normalization with the following equation: intracellular proliferation at specific time points* *= CFU of fungi at 2, 4 or 24 h after the 0 h time-point/CFU of fungi after phagocytosis at the 0 h time-point. Macrophage intracellular killing activity is inversely correlated with the number of yeast colonies (intracellular proliferation).

### Statistical analysis

The mean ± SE was used for data presentation, and the differences among groups were examined for statistical significance using the unpaired Student’s t-test or one-way analysis of variance (ANOVA) with Tukey’s comparison test for the analysis of experiments with 2 and 3 groups, respectively. The repeated measures ANOVA with Bonferroni post hoc analysis was used for the analysis of the data with several time-points. Survival analyses were evaluated with the log-rank test. *P* values* *< 0.05 were considered statistically significant. SPSS 11.5 software (SPSS Inc., Chicago, IL, USA) was used for all statistical analysis.

## Additional Information

**How to cite this article**: Surawut, S. *et al*. The role of macrophages in the susceptibility of Fc gamma receptor IIb deficient mice to *Cryptococcus neoformans.* *Sci. Rep.* **7**, 40006; doi: 10.1038/srep40006 (2017).

**Publisher's note:** Springer Nature remains neutral with regard to jurisdictional claims in published maps and institutional affiliations.

## Supplementary Material

Supplementary Data
