# Supplementary material for: The role of macrophages in the susceptibility of Fc gamma receptor IIb deficient mice to Cryptococcus neoformans
Source: Sci Rep. 2017 Jan 11;7:40006. doi: 10.1038/srep40006 (PMC5225418; doi:10.1038/srep40006)
Supplement: Supplementary Data [file srep40006-s1.pdf]

Manuscript title: The role of macrophages in the susceptibility of Fc gamma receptor IIb deficient mice to *Cryptococcus neoformans*

Author list: Saowapha Surawut, Thunnicha Ondee, Sujittra Taratummarat, Tanapat Palaga, Prapaporn Pisitkun, Ariya Chindamporn, Asada Leelahavanichkul

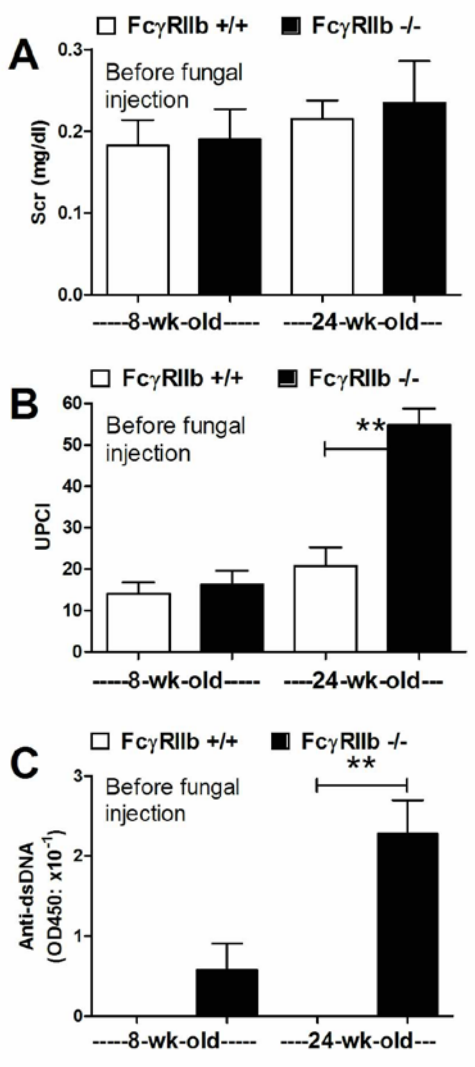

Supplementary Figure S1. Characteristics of FcγRIIb-/- mice at 8- and 24-weeks-old as demonstrated by serum creatinine (Scr) (A), proteinuria by urine protein creatinine index (UPCI) (B) and anti-dsDNA (C), (n=4-5/group). The data are shown as the mean ± SE, and the presented values are combined from 2-3 independent experiments. ** p<0.05, ** p<0.01.*

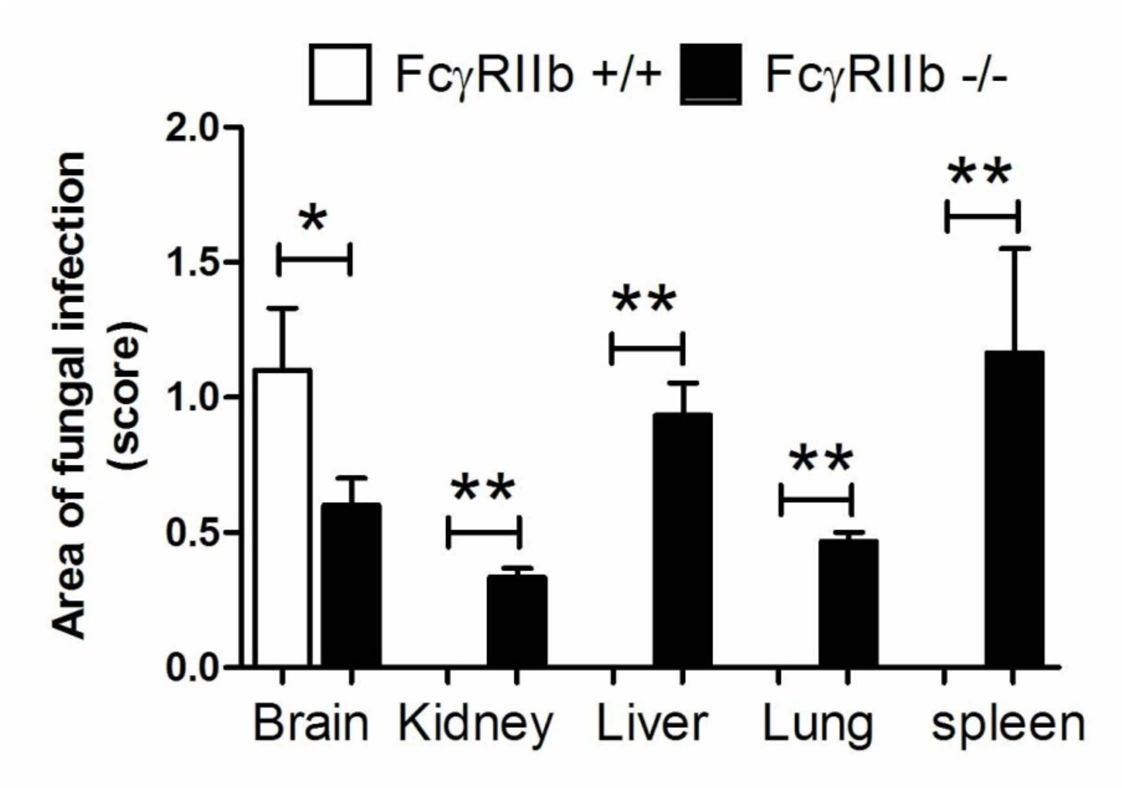

Supplementary Figure S2. The quantitative histology scoring of Figure 2 is graphically demonstrated (n=4-5/group). ** p<0.05, ** p<0.01.*

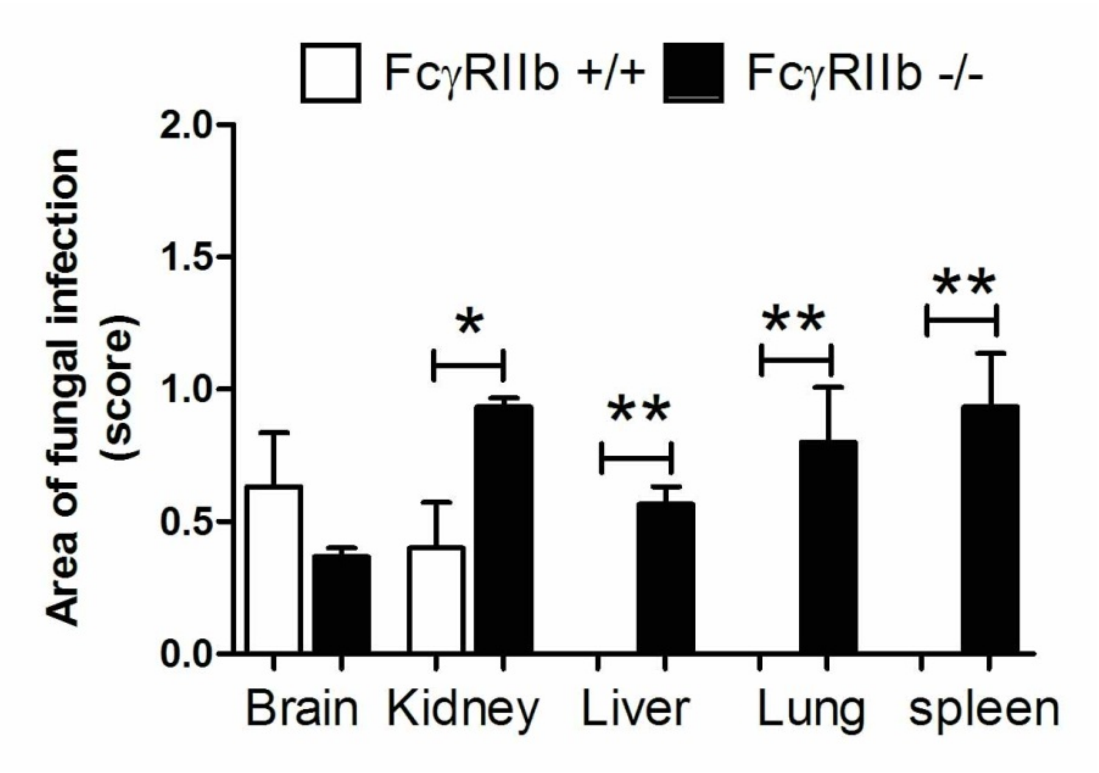

Supplementary Figure S3. The quantitative histology scoring of Figure 5 is graphically demonstrated (n=4-5/group). ** p<0.05, ** p<0.01.*

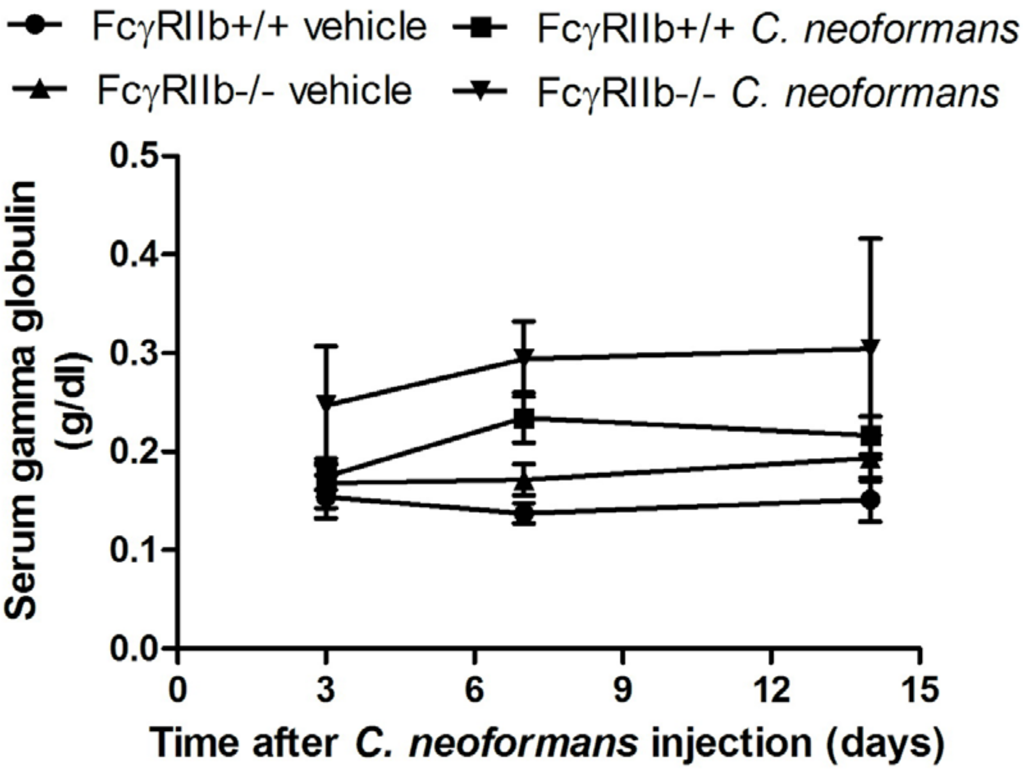

Supplementary Figure S4. The analysis of gamma globulin protein from capillary protein electrophoresis after *C. neoformans* injection is shown (n=4-6/group, the experiment was performed in 2 independent experiments). * $p<0.05$.

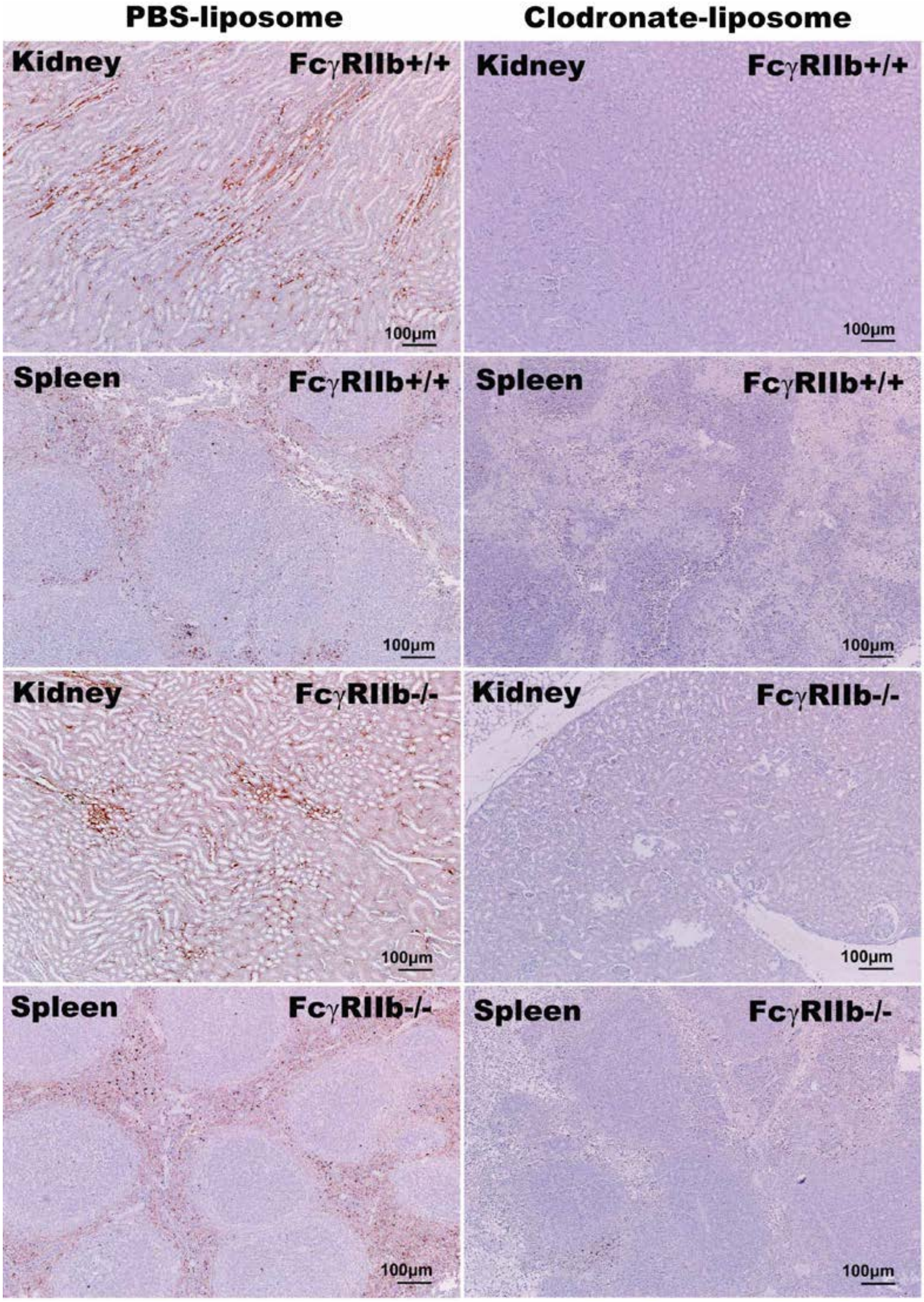

Supplementary Figure S5. Representative immunohistochemistry analysis of macrophages stained with F4/80 from FcγRIIb+/+ and FcγRIIb-/- with and without clodronate administration is shown. (Only spleens and kidneys were selected as representative organs)

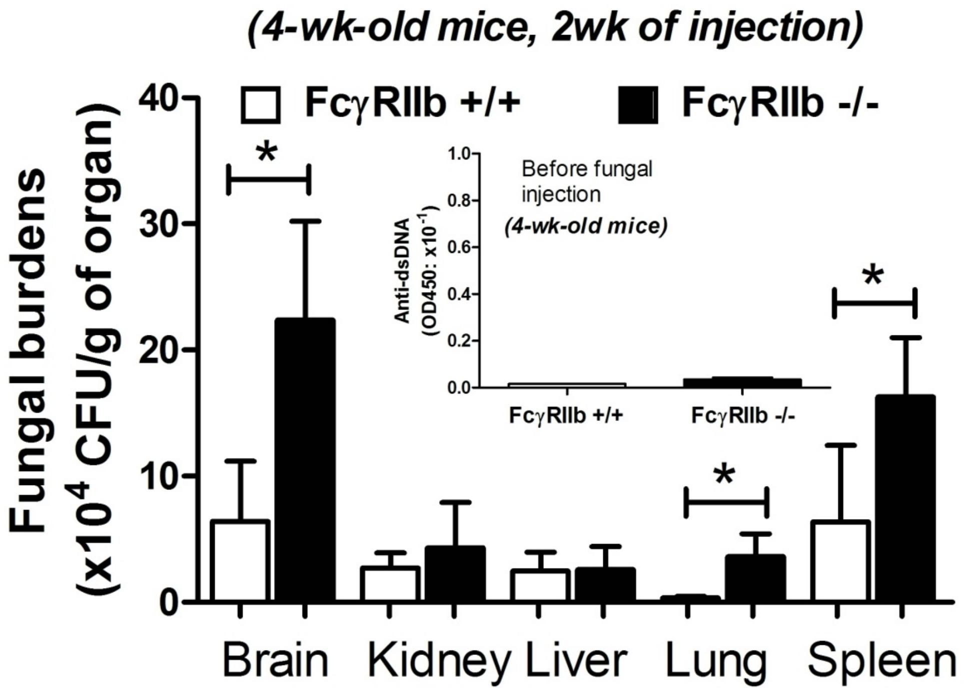

Supplementary Figure S6. Fungal burdens in the internal organs of 4-week-old at 2 weeks after *C. neoformans* administration and anti-dsDNA of FcγRIIb+/+ and FcγRIIb-/- mice at 4-weeks-old. The data are shown as the mean ± SE (n = 4-5/group). * $p<0.05$.
